# Double-antibody-based nano-biosensing system for the onsite monitoring of SARS-CoV-2 variants

**DOI:** 10.1038/s41378-023-00578-0

**Published:** 2023-08-21

**Authors:** Heba A. Hussein, Ahmed Kandeil, Mokhtar Gomaa, Rabeay Y. A. Hassan

**Affiliations:** 1 Virology Department, Animal Health Research Institute (AHRI), Agricultural Research Center (ARC), Giza, 12619 Egypt; 2 Biosensors Research Laboratory, Zewail City of Science and Technology, 6th October City, Giza, 12578 Egypt; 3 Center of Scientific Excellence for Influenza Viruses, Environmental Research Division, National Research Centre, Giza, 12622 Egypt; 4 Nanoscience Program, University of Science and Technology (UST), Zewail City of Science and Technology, Giza, 12578 Egypt

**Keywords:** Environmental, health and safety issues, Chemistry

## Abstract

The fast and reliable diagnosis of COVID-19 is the foremost priority for promoting public health interventions. Therefore, double-antibody-based immunobiosensor chips were designed, constructed, and exploited for clinical diagnosis. Gold nanoparticles/tungsten oxide/carbon nanotubes (AuNPs/WO_3_/CNTs) were used as the active working sensor surface to support the chemical immobilization of a mixture of SARS-CoV-2 antibodies (anti-RBD-S and anti-RBD-S-anti-Llama monoclonal antibodies). The morphology and chemical functionalization of the fabricated disposable immunochips was characterized using scanning electron microscopy (SEM), Fourier transform infrared (FTIR) spectroscopy, cyclic voltammetry (CV), and electrochemical impedance spectroscopy (EIS). After full assay optimization, the immunobiosensor showed a high sensitivity to detect SARS-CoV-2-S protein with limits of detection and quantification of 1.8 and 5.6 pg/mL, respectively. On the other hand, for the SARS-CoV-2 whole virus particle analysis, the detection and quantification limits were determined to be 5.7 and 17 pg/mL, respectively. The biosensor showed a highly selective response toward SARS-CoV-2, even in the presence of influenza, nontargeting human coronaviruses, and Middle East respiratory syndrome coronavirus (MERS-CoV). The immunochips exhibited distinct responses toward the variants of concern: B.1>C.36.3>Omicron> Delta> Alpha coronavirus variants. For biosensor validation, twenty-nine clinical specimens were analyzed, and the impedimetric responses were positively detected for two Delta samples, eighteen Omicron samples, and six B.1-type samples in addition to three negative samples. Eventually, the immunobiosensor was fabricated in the form of ready-to-use chips capable of sensitive detection of virus variants, especially variants of concern (VOC) and interest, in a specimen within 15 min. The chips provided instantaneous detection with the direct application of clinical samples and are considered a point-of-care device that could be used in public places and hot spots.

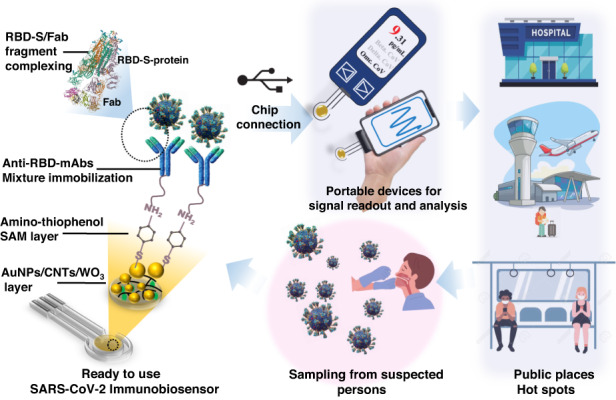

## Introduction

SARS-CoV-2 has been the third coronavirus to take an extraordinary toll on public health in the last two decades, after SARS-CoV and MERS-CoV in 2003 and 2012, respectively. Beta-coronavirus has a single-strand positive-sense RNA genome ~30,000 bp in length^1,2^. The virus is distinguished by the evolution of one or multiple genetic mutations.

Since the COVID-19 pandemic, different genetic circulating, emerging, and adaptive evolution variants of SARS-CoV-2 have struck worldwide^3^. The public health organizations categorized the variants on the basis of the viral spread among countries, the public health risk, and the recorded substitutions in the spike protein that influence the host monoclonal antibody response, replacement from one variant to another among the populations, and the variant dominance. Currently, the variants being monitored include Alpha (B.1.1.7 and Q lineages), Beta (B.1.351 and descendent lineages), Delta (B.1.617.2 and AY lineages), Gamma (P.1 and descendent lineages), Epsilon (B.1.427 and B.1.429), Eta (B.1.525), Iota (B.1.526), Kappa (B.1.617.1), Mu (B.1.621, B.1.621.1), B.1.617.3, Zeta (P.2), and the recently emerging Omicron (B.1.529, BA.1, BA.1.1, BA.2, BA.3, BA.4 and BA.5) SARS-CoV-2 lineages. Alpha, Beta, Gamma, Delta, and Omicron SARS-CoV-2 lineages have been classified as variants of concern (VOCs)^4,5^.

Spike protein (S protein) engages the cellular integrin angiotensin-converting enzyme 2 (ACE-2) through the viral receptor-binding domain (RBD) to invade susceptible host cells. Moreover, the genetic mutations of the variants form hallmarks in the amino acid sequence of the SARS-CoV-2-S protein, especially the RBD, and each has varying numbers of substitutions in the N-terminal domain^5^.

Notably, most of clinically used antibodies lost efficacy and affinity toward most variants, especially the current Omicron variants, due to a large number of mutations: >30 substitutions, insertions, and deletions^6^. Genetic changes have implications in the immunogenic response to viral infection, vaccination, and therapeutic regimes, and the population could therefore be jeopardized by the emergence of new variants^7,8^. Thus, the accurate and effective diagnosis of the virus is crucial for curtailing the disease spread among populations. In this regard, miniaturized immunobiosensors using anti-SARS-CoV-2 antibodies were deployed to overcome expected issues regarding the authorized diagnostic procedures and find a path toward feasible point-of-care technologies^9,10^.

Gold nanoparticles have been used extensively in various biosensing technologies, including surface plasmon resonance and colorimetric, electrochemical, or dual-purpose biosensors^11^. For instance, a dual-functional plasmonic biosensor based on photothermal effects and localized plasmon resonance was established using two-dimensional gold nanoislands, where the NI surface was functionalized with complementary DNA that was subsequently hybridized with the viral nucleic acid. Upon the application of plasmonic resonance frequency, thermoplasmonic heat was generated on the chip to increase the temperature of the in situ hybridization and subsequently enhance the chip identification of genes and multigenes at a detection limit of 98 pg/mL^11^.

In another study, for the selective recognition of S protein, ACE2 portion was conjugated onto a surface modified with gold nanoparticles and graphene. This setup was integrated into a homemade portable potentiostat and connected to a smartphone for the rapid monitoring of targeting antigens. The detection limits were 5.14 and 2.1 ng/mL for the S1 and S2 proteins, respectively. Accordingly, 63 clinical samples of Alpha, B.1, and Delta variants were investigated^12^. In addition, a carboxylic-rich dual-functional electrode was functionalized with anti-nucleocapsid antibodies to detect nucleoproteins. The detection limits were 116, 150 fg/mL for the spiked and nasopharyngeal samples, respectively^13^. Moreover, a graphite pencil was used for the detection of S protein when ACE-2 was immobilized on its surface using a glutaraldehyde/AuNP/cystamine matrix^14^. The results of this study demonstrated that high sensitivity to B.1.1.7 UK variants was achieved with a limit of detection of 229 fg/mL. In another study, S protein was detected using an electrochemiluminescence immunosensor approach that was developed with Au@BSA-luminal nanocomposites^15^.

Electrochemically derived biosensing techniques provide reliable, miniaturized, and on-site portable analytical devices. In particular, electrochemical biosensors were developed for the detection of SARS-CoV-2 (Table 1), including a 30-min developed sandwiching immunoassay proposed for the detection of SARS-CoV-2 S and N proteins. The enzymatic reaction was investigated to detect the byproduct 1-naphthol using screen-printed electrodes modified with carbon black nanomaterials on the bases of monoclonal anti-S or anti-N protein-functionalized magnetic beads and secondary antibodies with alkaline phosphatase as a label. The analytical sensitivity was 19 ng/mL and 8 ng/mL for both S and N proteins, respectively^16^.

**Table 1:** A short list of electrochemical biosensors developed and used for SARS-CoV-2 detection

Sensor	Detection Technique	Biosensor platform	Functionalization biomarkers	Sensor base	Sample	Detection time	Limit of detection (LOD)	Linear range	References
Immunobiosensor	DPV	Sandwich label based MBs/mAbs anti-S/anti-N/Secondary antibodies-Alkaline phosphatase/Carbon black SPE	mAbs-S protein mAbs-N protein	CB-modified SPEs	Saliva	30 min	19 ng/mL 8 ng/mL	0.04–10 μg/mL 0.01–0.6 μg/mL	^16^
Immunobiosensor	CV	BSA/AB/*f*-GO/GCE BSA/AB/*f*-GO/SPE	Anti-SARS-CoV-2-S antibodies	*f*-GO-GCE *f*-GO-SPE	Saliva and oropharyngeal swabs	35 min 5 min	1 ag/mL	1ag–10 fg/mL	^17^
Immunobiosensor	CV	Graphene/Anti-S antibodies/SPE	Anti-SARS-CoV-2-S antibodies	Graphene	Spiked samples	45 min	20 µg/mL	20–80 µg/mL	^18^
Immunobiosensor	SWV	NHS/EDC/SPE/Carbon nanofibers/Anti-N antibodies	Anti-SARS-CoV-2-N antibodies	Carbon nanofibers	Spiked nasal samples	20 min	0.8 pg/mL	1 pg–1000 ng/mL	^19^
Immunobiosensor	Voltammetry	Graphene/Antisense ssDNA oligonucleotide-modified AuNPs/Paper based	Antisense-ssDNA-SARS-CoV-2 N gene	Graphene	Virus-infected Vero cells and clinical samples	5 min	6.9 copies/μL	10^3^–10^8^ copies/μL	^30^
MIP based	DPV	nCoV-NP-MIP/Au-TFE	nCoV-NP-MIP	Au-TFE	Nasopharyngeal swabs	5 min	7 pg/mL	1–49 pg/mL	^20^
RNA biosensor	EIS	4-Amino thiophenol (4-ATP)/EDC/NHS	RNA	AuNPs/WO_3_	Not applicable	45 min	0.13 ng/mL	0.0045 ng/mL–µg/mL	^3^
MIP based	EIS	CNTs/WO_3_-modified SPE/Whole-virus imprinting in mAP polymer matrix	Created complementary binding sites of SARS-CoV-2 whole virus	CNTs/WO_3_	Nasopharyngeal swabs	5 min	57 pg/mL	7–320 pg/mL	^21^
Immuno-biosensor	EIS	AuNP/WO_3_/CNT-based SPE/ATP/mixture of Anti-SARS-CoV-2-S antibodies	A mixture of mAbs-S-anti-Llama antibodies mAbs-S-antibodies	CNTs/WO_3_/CNTs	Nasopharyngeal swabs	15 min	1.8 pg/mL (S protein) 5.7 pg/mL (Whole virus)	0.125 fg–16.0 pg/mL 0.01–74 pg/mL	This work

*DPV* differential pulse voltammetry, *MBs* magnetic beads, *mAbs* monoclonal antibodies, *S protein* spike protein, *N-protein* nucleoprotein, *SPE* screen-printed electrode, *CV* cyclic voltammetry, *BSA* bovine serum albumin, *AB* antibody, *f-GO* functionalized graphene oxide, *ag* attogram, *fg* femtogram, *pg* picogram, *NHS* N-hydroxysuccinimide, *EDC* carbodiimide, *Au-TFE* gold thin-film electrode, *MIP* molecular imprinted polymer, *mAP* meta-aminophenol, *ATP* aminothiophenol, *WO*_*3*_ tungsten oxide, *CNTs* carbon nanotubes

In another reported study, anti-SARS-CoV-2-S protein antibodies mounted on graphene oxide-modified electrodes were used for the analysis of S protein^17^. A graphene-based label-free immunoassay was developed using anti-S protein antibodies for the detection of the virus. The electrochemical reaction is mediated by [Fe(CN)_6_]^3−/4−^, where the S1 protein subunit was detected at 20 µg/mL^18^. On the other hand, immobilization of N protein was conducted on screen-printed electrodes modified with carbon nanofibers and electrografted diazonium surfaces. This sensor provided a detection limit of 0.8 pg/mL^19^.

The molecular imprinting of SARS-CoV-2 N protein was exploited to construct molecularly imprinted polymer-based biosensors, and the detection and quantification limits of the antigen were 7 and 22 pg/mL, respectively^20^. In addition, virus-imprinted biosensors were developed using CNT/WO_3_-modified microchips and exploited for the rapid detection of the whole SARS-CoV-2 virus in clinical samples through complementary binding pockets created for the virus on the imprinted chips. The- fabricated chips were highly sensitive at a detection limit of 57 pg/mL, estimated to be 27-fold more sensitive than RT‒PCR, and high selectivity was obtained^21^.

Because of their signal amplification properties, the larger surface area for biomolecule immobilization, and subsequently increased binding sites to detect the analyte, nanomaterials are intensively employed in biosensing technologies^3,10^. Carbon nanotubes (CNTs) possess high electronic conductivity and promote electron transfer and electrochemical stability in a variety of solutions. Gold nanoparticles have been used extensively in the development of different biosensors. Metal oxides have been widely applied in biosensing, catalysis, biomedicine, energy storage, and automobile catalytic converters. Among various metal oxides, tungsten oxide (WO_3_) is used in many biosensor and electronics applications of due to its chemical stability, biocompatibility, and catalytic activity^22^. Therefore, the newly developed double antibody-based immunosensor is supported by the functionalization of disposable printed electrodes with a nanocomposite (AuNPs/WO_3_/CNTs) to provide effective chemical immobilization of a mixture of SARS-CoV-2 antibodies.

## Material and methods

### SARS-CoV-2 propagation, clarification, and concentration

The isolate used was propagated from the recorded (hCoV-19/Egypt/NRC-03/2020 SARS-CoV-2 strain (GISAID accession number: EPI-ISL-430820)). Briefly, the virus inoculum was propagated in a Vero-E6 cell line for 1–3 days of incubation at 37 °C in a 5–6% CO_2_ incubator. Afterward, the virus suspension was clarified twice for 30 min at 4500 rounds/min. Using 20% sucrose, the suspension was ultracentrifuged at 50,000 × *g* for 1.0 h at 4.0 °C. The antigenic payload was determined using a NanoDrop 2000c at an absorbance wavelength of 280 nm. The concentrated antigen was stored at −80 °C until further use.

### Sensor surface modification with nanomaterials

First, aqueous dispersions (5.0 mg/mL) of nanomaterials were prepared. Then, screen-printed electrodes were modified with gold nanoparticles (AuNPs) alone or with AuNPs/metal oxide nanocomposites including selenium oxide (SeO_2_), manganese dioxide (MnO_2_), tungsten oxide (WO_3_), germanium oxide (GeO_2_), zirconium oxide (ZrO_2_), and cerium oxide (CeO_2_). Subsequently, highly electrochemically active AuNP/metal oxide composites were tested after twinning with multiwall carbon nanotubes (10 mg/mL) (AuNP/metal oxide/CNT composites). The nanomaterials were loaded on the 3.0 mm plain carbon screen-printed electrodes as active working electrodes. The modified electrodes were electrochemically tested in 5.0 mM ferricyanide (FCN) as the standard redox probe.

### Anti-SARS-CoV-2 monoclonal antibody immobilization

Two fragments of antibodies, SARS-CoV-2 spike RBD antibody (a monoclonal mouse IgG_2B_ clone #1034522, Cat. No.: MAB10540-100, R&D systems-biotech) and SARS-CoV-1/2-Spike-RBD-LlaMABody (a recombinant monoclonal human IgG1 clone # VHH72, Cat. : LMAB10541-100, R&D systems-biotech) were used to fabricate SARS-CoV-2 immunobiosensor chips. A mixture of anti-S and anti-Llama antibodies (1.0 and 2.0 µg/µL, respectively) was immobilized on the nanomodified electrode. First, the electrodes were activated and functionalized by a layer of 4-aminothiophenol (4-ATP). The electrodes were incubated overnight in a solution of 4-ATP monomers (0.3 M) at 4 °C and then washed with ethanol to remove the nonassembled ATP molecules from the surface^23,24^. Consequently, the 4-ATP-based electrodes were incubated in a solution of mixed SARS-CoV-2 antibodies for 12 h at room temperature. The immunobiosensor chips were thoroughly washed with PBS (pH = 7.4) to flush away nonimmobilized antibodies. The ready-to-use biosensors were preserved at 4 °C until use.

### Electrochemical measurements

Using a redox-mediated electrochemical system, the electrochemical characteristics of the newly designed immunobiosensor were identified, optimized, and applied for the detection of the target virus. For this purpose, EIS measurements were conducted at an AC potential of 0.005 V, applied frequency range of 10.000 to 0.1 Hz, and applied DC potential of 0.4 V. Cyclic voltammetry (CV) analysis was carried out at a scan rate of 0.05 V/s, and the potential ranged from -0.2 to 1.0 V versus Ag/AgCl. All electrochemical tests were performed in potassium FCN as a standard redox probe with a concentration of 5.0 mM. All electrochemical measurements were carried out using a PalmSens4 device.

### Randles impedimetric circuit design

For the quantitative data analysis and Nyquist plot EIS data interpretation, a specific Randles electronic (equivalent) circuit was simulated. The equivalent circuit included a solution resistance (R1), a capacitance layer of AuNP/WO_3_/CNT nanocomposite on the sensor interface (C1), the diffusion resistance of the redox probe displayed in Warburg impedance (W1), a charge transfer resistance (R2), and a constant phase element of the sensor inner layer of the self-assembly monolayer of amino-thiophenol (CPE1), which models the double-layer capacitor of the sensor surface. Ultimately, the resistance element of SARS-CoV-2-S protein binding with the immobilized mAbs is expressed in R3. The obtained electrochemical signals of the immunosensors were fitted using the modeled electrical circuit to extract the values of all generated resistances.

### Immunobiosensor performance testing

The immunobiosensor performance was tested with different concentrations of Wuhan-like SARS-CoV-2 S protein (Recombinant SARS-CoV-2 Spike RBD FC Chimera, Cat. : 10499-CV-100, R&D systems-biotech). Seven concentrations of the spike protein (ranging from 0.125 pg to 16.0 pg/mL) were used to test the binding efficiency of the immobilized mAbs toward the target RBD of the S protein. Moreover, the sensing performance of the prepared sensor chips was tested against whole SARS-CoV-2 particles at a wide range of virus concentrations (from 0.01 to 74 pg/mL). The binding time for the immunogenic reactivity between the antigen and the antibody mixture was tested in a period ranging from 5 to 75 min at room temperature, and the experiments were carried out using a single concentration of the recombinant S protein (0.02 ng/mL).

### Selectivity testing

The impedimetric response of the immunobiosensor was tested against nontarget viruses, including influenza A and B, human coronaviruses (hCoVs) OC43, NL63, and 229E, and Middle-East Respiratory Syndrome Coronavirus (MERS-CoV). A suspension of each virus was tested individually on the designed immunochips. Furthermore, mixtures of multiple viruses including and excluding the target SARS-CoV-2 were tested for confirmatory validation. The electrochemical responses were compared to those of the target virus.

### Testing the response of the fabricated chips to existing variants

The selectivity of the newly developed immunobiosensor was tested against various recorded variants. Virus suspensions (10 µL) of Alpha, B.1, Delta, Omicron, and C.36.3 lineages with concentrations of 1.02, 0.7, 0.6, 0.4, and 0.6 ng/mL, respectively, were drop-cast on the prepared immunochips and incubated for 15 min at room temperature for subsequent electrochemical investigations. Then, the impedimetric responses were recorded and analyzed using the modeled equivalent circuit.

### Clinical sample analysis

With no sample treatments, collected nasopharyngeal swabs were directly dropped on the prepared immunochips. The specimens were collected from patients in a virus-transport medium. A 5.0 µL aliquot of each of the collected thirty-three samples was used on the prepared chips. Sample-loaded chips were incubated for 15 min at room temperature. Afterward, the chips were rinsed with PBS to remove any nonreacted particles. Electrochemical signals of each sample were collected three times, and both positive (i.e., standard virus concentration) and negative controls were included for validation and confirmation.

### Sensor validation using rT-RT‒PCR

In parallel to the electrochemical measurements, the tested clinical samples were analyzed using RT‒PCR as the authorized diagnostic method. The procedures were applied as per the authorized protocol by Chu et al. ^25^. In brief, for viral RNA extraction, a QIAamp Viral-RNA Kit (Qiagen, Germany) was used. Genome amplification was performed with a Verso 1-Step qRT‒PCR Kit (Thermo, USA) by using specific primers and probes (Open Reading Frame 1ab (ORF1ab)-nsp14 gene assay). In a tube, 25 µL of the total reactant solution was prepared containing 1.0 µL of 10 µM primers, 5.0 µL of the extracted template, 0.5 µL of 10 µM probe, 1.25 µL of RT-enhancer, 0.25 µL of reactive enzyme mixture, and 3.5 µL of ddH_2_O, and then the reactant volume was completed by adding 12.5 µL of the one-step buffer. The amplification was carried out for 45 cycles of reverse transcriptase and polymerase activation at 50 and 95 °C for 15 min. The denaturation step was 15 s at 95 °C throughout. Subsequently, annealing and extension steps were conducted at 60 °C for 30 s. An in-house synthetic plasmid was designed as a positive control. The results were analyzed via the designated threshold line (CT), and the copy number was estimated. All clinical samples were collected and analyzed by Virology Department members at the National Research Centre (NRC, Cairo, Egypt).

### Data analysis and statistics

The obtained data were expressed and presented as the mean ± SD from three individual measurements. The statistical significance was estimated by the statistical hypothesis, and the significance of the obtained values was assumed to be *p* < 0.05. Limit of detection, LOD = (3.3 × SD)/slope of the curve, and limit of quantification, LOQ = (10 × SD)/slope of the curve were calculated in correlation to the designed calibration curve. The reproducibility of the immunobiosensor was presented as the relative standard deviation. All the electrochemical signals were plotted as correlated figures, and the values were analyzed using Origin-Lab software.

## Results and discussion

### Sensor surface modification with nanomaterials

**Fig. 1:**
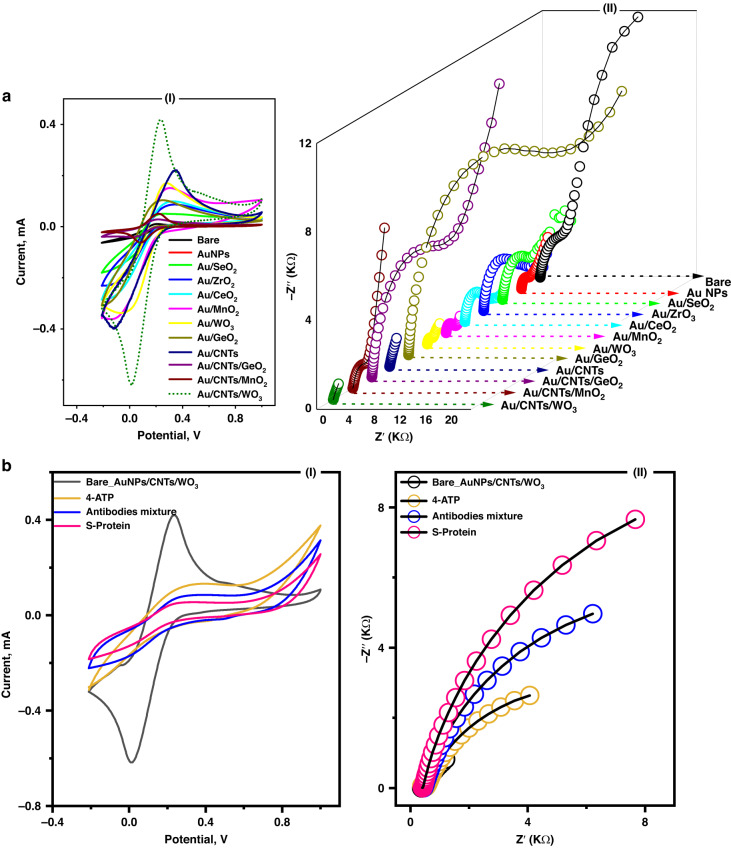
Fabrication,characterizations, and testing the electrochemical performance of the newly developed nano-biosensor. **a** (I)**-**Electrochemical characterization of metal/metal-oxides/CNTs modified electrodes using cyclic voltammetry (CV) at the scan rate of (50 mV/s), and potential range (−0.2 to 1.0 V). (II)**-**Electrochemical impedimetric spectroscopy (EIS) was conducted within the frequency (10,000 to 0.1 Hz) and the applied DC of +0.4 V. FCN (5 mM) was used as the standard redox probe. **b** Voltammetric and impedimetric characterizations (I and II, respectively) of the immune-sensor surface changes including the surface functionalization with the cross-linking agents (4-ATP), and a mixture of SARS-CoV-2 antibodies

A successfully designed high-performance electrochemical biosensor is usually highly conductive and highly electroactive with an expandable surface area. Thus, screen-printed electrodes were modified with various nanostructures, including metals, metal oxides, carbon nanotubes, or nanocomposites of those nanomaterials. The electrochemical responses of each modified electrode were tested electrochemically using CV and electrochemical impedance spectroscopy (EIS). In a large screen, electrode modifications with gold/metal oxide (GeO_2_, WO_3_, and MnO_2_) nanocomposites exhibited the highest electrochemical signals, as shown in Fig. 1a. Moreover, the electrochemical signals were further improved when carbon nanotubes were integrated into the Au/metal oxide nanocomposite. Eventually, AuNP/WO_3_/CNT-modified screen-printed electrodes (10/5.0/10 mg/mL, respectively) were selected due to their synergistic electromechanical responses (almost fourfold higher than the untreated electrode, Fig. 1a).

### Immobilization of SARS-CoV-2 antibody mixture

A mixture of two antibodies (SARS-CoV-2 Spike RBD antibody and SARS-CoV-1/2 Spike RBD Llamabody antibody) was selected for the fabrication of the SARS-CoV-2 biosensor. The use of a combination of antibodies (IgG_1_ and IgG_2B_ isomers) grants high-affinity binding to the target S protein of the virus^25^. The two isomers are produced in the human body, and the hinge of IgG_2B_ is shorter by 12 amino acids than that of IgG1. The isomere (IgG_2B_) is quite different in the number of disulfide bridges in the hinge region, which provides conformational flexibility of the Fab portion.

Therefore, combined functionalization with both antibodies provides high and flexible selective detection of S protein with high orientation toward the target S protein. After selecting the nanomaterials for the platform to support the immobilization of the SARS-CoV-2 mixture-antibodies, a self-assembled monolayer of 4-amino thiophenol (4-ATP) was first formed as a cross-linker chain to orient the covalent immobilization and self-assembly of the targeting antibodies. The resulting thin film of the 4-ATP monolayer provided bifunctionalized active (-NH2)-groups that oriented perpendicular to the electrode surface through thiolation with homogeneously dispersed gold nanoparticles. Thus, the selected antibodies are covalently bonded to the terminal end of the SAM layer through their free carboxylic group (i.e., -COOH of the FC portion), and this chemical immobilization creates antigen/antibody binding sites. Electrochemical characterizations were carried out at each step of the sensor preparation and functionalization, and the results are presented in Fig. 1b, showing that the change in the electrochemical signals (CV or EIS) was dependent on the surface composition and its matrix. The blocking of redox reactions, reflected by the voltammetric and impedimetric signals, indicated stable antibody immobilization and full coverage of the sensor surface with the maximum capacity for antibody loading.

### Immunobiosensor chip characterization

Functional and morphological characterizations were directly conducted on the sensor chips to determine the chemical and physical changes that resulted from the nanomaterial modifications, 4-ATP cross-linking functionalization, and antibody immobilization. For functional analysis of the sensor surface, the Fourier transform infrared (FTIR) technique was used: the disposable chips were investigated directly using an FTIR-diamond probe within the region of 1660–740 cm^−1^. The self-assembled monolayer of 4-ATP showed a band at 1083 cm^−1,^ which was assigned to the C=S (thiocarbonyl) stretching vibration. The C-C stretching vibration of the benzene rings was detected at the rational band of 1592 cm^−1^. It is worth mentioning that three stretching bands that appeared at regions 1175, 1411, and 1592 cm^−1^ were assigned to the perpendicular coupling of the formed 4-ATP to the nanomodified surface.

**Fig. 2:**
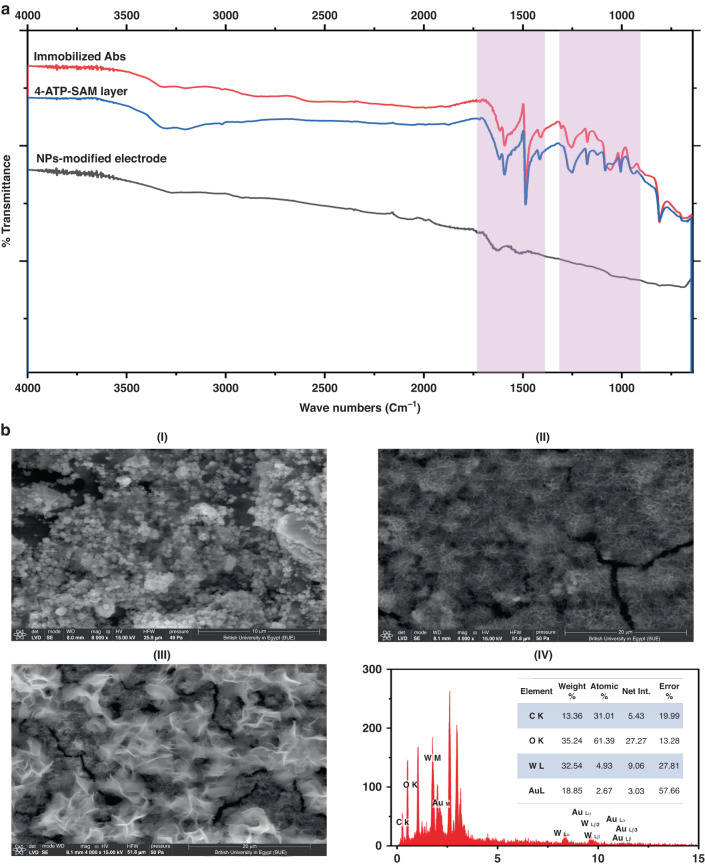
Structural and morphological identification of the biosensor surface. **a** Fourier transform-Infrared (FTIR) characterization of the nanocomposite-modified electrode, after layering with the 4-ATP, and antibodies immobilization. **b** Scanning Electron Microscopy (SEM) images of the immuno-biosensor fabrication steps: (I) image of the nano-modified surface, (II) the self-assembled monolayer of the 4-ATP, (II) immobilization of the anti-SARS-CoV-2 antibodies. (IV) The elemental mapping by Energy Dispersive X-ray (EDX) of the AuNPs/CNTs/WO_3_ layer with the remarkable expression of the AuNPs, WO_3_, and CNTs on the electrode surface. The inset represents the percentage of the elemental distribution

On the other hand, the immobilization of the antibodies on the 4-ATP layer was identified through several detected stretching and vibrational bands at 1084 cm^−1^, 1252 cm^−1^, 2054 cm^−1^, 1618, and 1593 cm^−1^, which were assigned to the functional groups NH_2_, C-N, -N=C=O, and C-N, respectively, as shown in Fig. 2a.

For the morphological and surface composition analysis, SEM-EDX imaging and elemental analysis were performed after surface modification with the nanomaterials, cross-linking with 4-ATP, and loading with antibodies, as shown in Fig. 2b. SEM images of the modified screen-printed electrodes showed that the nanoparticles were well distributed and covered the electrode surface (I). Figure 2b-II and III illustrate that the changes resulting from sensor surface modification with the cross-linker as well as the antibody can be seen clearly. Surface composition analysis and elemental mapping obtained by energy dispersive X-ray (EDX) showed the percentage elemental distribution on the working area of the electrode: carbon, tungsten, and gold occupied 14%, 33%, and 19%, respectively, of the electrode area (IV).

### Immunobiosensor assay optimization

**Fig. 3:**
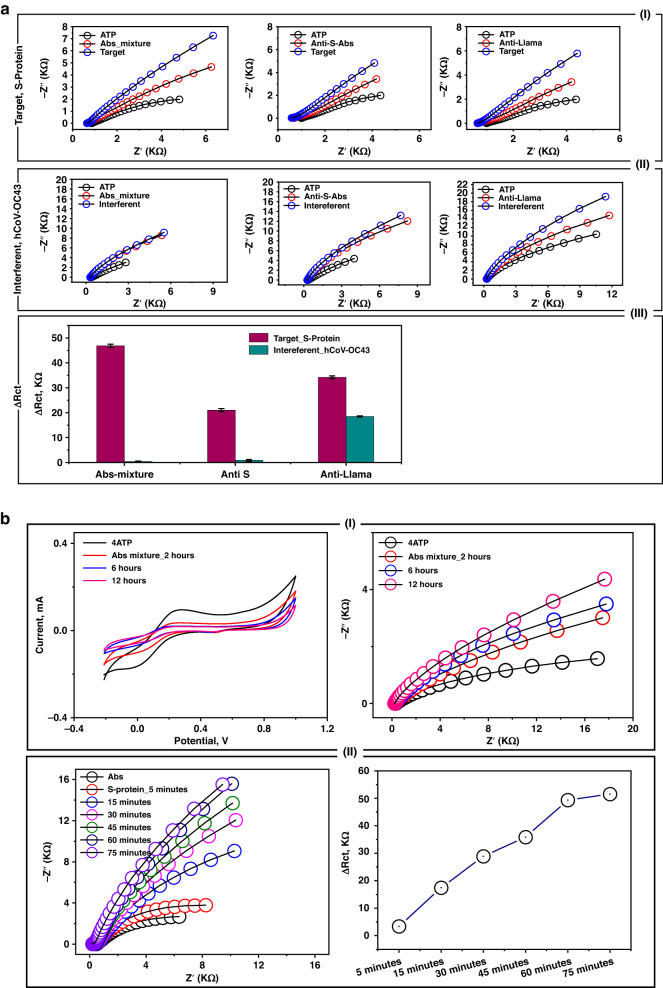

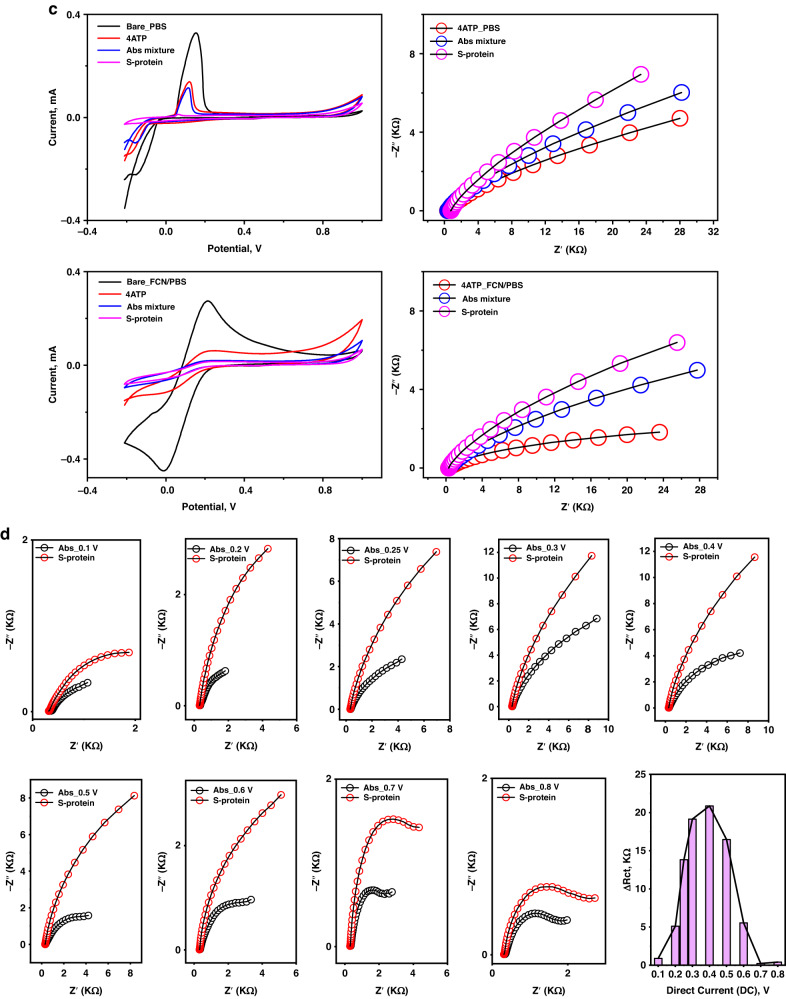
Electrochemical steps for assay optimizations. **a** (I)**-**Testing the impedimetric performance of single antibody-based biosensor (SARS-CoV-2 Spike RBD antibody, or SARS-CoV-1/2 Spike RBD Llamabody antibody), or double antibody-based biosensor (a mixture of two antibodies). The EIS signal was individually measured for each sensor before and after capturing the targeting antigen (SARS-CoV-2-S-protein). (II)-Primary selectivity testing using the fabricated immuno-biosensor towards an interferent virus (hCoV-OC43). (III)-Bar curve demonstrating the difference of the electrochemical response values (Δ*R*_ct_, kΩ) to show the binding capacity of the mixture of SARS-CoV-2 antibodies (Anti-S and Anti-Llama) wit the target (S-Protein). **b** (I)**-**CV and EIS measurements for testing the time of antibodies immobilization from 2.0 to 12 h. (II)**-**EIS testing for different antigen-antibody binding/interaction time intervals (from 5.0 to 75 min). **c** EIS measurements for testing the immuno-biosensor performance in non-mediated (PBS) or mediated (FCN) electrochemical systems. The measurements were conducted at +0.4 V. **d** Effect of the applied direct current (DC) on the immuno-biosensor performance. The measurements were conducted in the mediated FCN system within the frequency range of (10,000 to 0.1 Hz). The bar figure indicated the difference in the electrochemical response (expressed as Δ*R*_ct_, kΩ) before and after the S-protein binding to the immobilized antibodies at different DC potential points

The immunosensor optimization and application were subsequently completed with EIS, as it can sensitively measure the selective and dynamic (antibody-antigen) binding events that occur at the sensor surface. Accordingly, different immunobiosensors were constructed using a single antibody (either anti-S or anti-Llama) or a mixture of the two. The impedimetric responses were individually tested against the targeting virus strain or another foreign (nontargeting) virus strain named hCoV-OC43. As shown in Fig. 3a, a strong binding affinity and capacity were obtained when the mixture of the antibodies was used as a sensing platform. The high selectivity for the target virus strain was clear since a very high impedimetric signal (47.8 kΩ) was received from the target antigen-antibody interaction, while a very weak response (0.37 kΩ) was collected from the interferent response. However, a severe cross-reactivity (interference problem) with the interferent strain was obtained when a single antibody was used as the sole biorecognition site, especially anti-Llama, as depicted in Fig. 3a-II.

The use of a combination of antibodies (IgG1 and IgG2B) grants high-affinity binding to the target S protein of the virus^26^. IgG2B is quite different in the number of disulfide bridges in the hinge region that provide conformational flexibility of the Fab portion. Therefore, combined functionalization with both antibodies provides high and flexible selective detection of S protein with high orientation toward the target S protein.

Consequently, the time of antibody immobilization onto the nanostructured surfaces was tested over a long duration (2, 6, 12, and 24 h). As shown in Fig. 3b-I, a minimum of 12 h was needed to reach full coverage of the sensors with the antibodies. Then, the antigen-antibody binding interaction time (the actual sensing time) was studied from 5.0 min to 75 min. The highest increase in the EIS signal was obtained after 30 min, establishing a strong and effective sensing time, as shown in Fig. 3b-II.

Regarding the optimization parameters that affect the EIS signal, mediated vs. nonmediated electrochemical measurements were conducted, and the effect of applied DC values was investigated. The standard redox mediator (FCN) enabled the highest EIS response compared with the nonmediated system, as shown in Fig. 3c. Accordingly, different DC values (from 0.1 to 0.8 V) were applied, and at each DC value, the EIS response of the antibody-antigen interaction was tested, as shown in Fig. 3d. From the Nyquist plots, the maximum difference in the charge transfer resistance (Δ*R*_ct_) was obtained when 0.4 V was applied. Thus, the FCN-mediated system with 0.4 V was selected for further investigation.

### Calibration curve

**Fig. 4:**
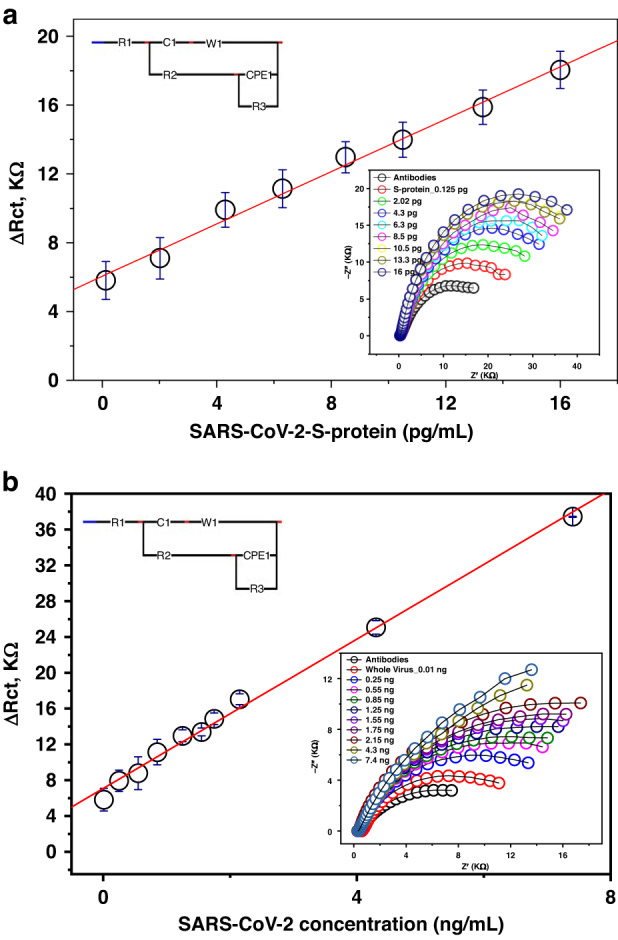
Sensitivity testing for the newly developed biosensors. **a** The calibration curve of the fabricated immuno-biosensor using different concentrations of S-protein ranged from 0.125 fg/mL to 16 pg/mL. **b** The calibration curve of the fabricated immuno-biosensor using different concentrations of whole SARS-CoV-2 particles ranged from 0.01 to 74 pg/mL. The resulting value is considered as a Δ*R*_ct_ value (*R*_ct_ virus complex−*R*_ct_ MAbs) which is estimated from the designed Randles impedimetric cell circuit

Different S protein concentrations ranging from 0.125 pg to 16 pg/mL were applied to the fabricated anti-S-RBD-based biosensor. The impedimetric signals were investigated as representative responses to the different antigen concentrations conjugated with the immobilized antibodies (Fig. 4a). According to the obtained calibration curve, a linear regression was obtained with a 0.995 *R*^2^-value, *P* < 0.0001, and the calculated limits of detection and quantification were 1.8 and 5.6 pg/mL, respectively.

In addition to this calibration curve, another calibration curve was obtained from the immunosensing responses with different concentrations of whole virus particles. In this experiment, complete virus particles replaced the pure S protein to validate the newly developed biosensor against raw and complex clinical samples. An additional standard curve was obtained, and the estimated limits of detection and quantification were 5.7 and 17 pg/mL, respectively, for SARS-CoV-2 whole virus particles (Fig. 4b). The proposed immunobiosensor showed reasonable analytical performance compared to the other electrochemical biosensors used for the detection of SARS-CoV-2, as given in Table 2.

**Table 2:** SARS-CoV-2 analysis in clinical specimens using the newly developed immuno-biosensor

Sample No.	Sample Origin	rT-RT‒PCR			Anti-RBD-based immunobiosensor		
		(Cycle Threshold-Ct)	Copy number/rxn	Typing	Δ*R*_ct_ (*R*_ct_ _virus_ − *R*_ct_ _antibodies_) kΩ ± SD	Antigenic payload (pg/mL)	Copy number/µL
S1	**Nasopharyngeal swab**	34	2129	Delta	7.6 ± 2.3	7.5 ± 2.3	68437
S2		32	9158	Delta	7.9 ± 3.1	9.2 ± 3.1	83949
S3		35	1382	Omicron	9.8 ± 2.7	12.5 ± 2.7	114061
S4		31	11917	Omicron	10.2 ± 2.0	12.3 ± 2.0	112236
S5		33	4102	Omicron	8.5 ± 2.4	10.1 ± 2.4	92161
S6		31	11917	Omicron	10.3 ± 3.4	13 ± 3.4	118623
S7		31	11917	Omicron	10.5 ± 1.8	13.5 ± 1.8	123186
S8		29	29317	Omicron	11.8 ± 1.4	16 ± 1.4	145998
S9		30	18964	Omicron	11.3 ± 3.2	18.6 ± 3.2	169723
S10		26	321567	Omicron	12.1 ± 3.4	18.2 ± 3.4	166073
S11		32	9158	Omicron	9.9 ± 1.7	12.5 ± 1.7	114061
S12		17	362874611	Omicron	35.4 ± 5.6	72.5 ± 5.6	661553
S13		30	18964	Omicron	11.4 ± 2.8	11.6 ± 2.8	105848
S14		18	352774511	Omicron	34 ± 2.7	65 ± 2.7	593117
S15		21	2754192	Omicron	26.3 ± 1.3	42.5 ± 1.3	387807
S16		30	18964	Omicron	11.5 ± 1.7	15 ± 1.7	136873
S17		25	1856421	Omicron	15.1 ± 1.9	27 ± 1.9	246371
S18		24	2541238	Omicron	19.5 ± 2.9	37.5 ± 2.9	342183
S19		26	321567	Omicron	12.6 ± 0.3	16.4 ± 0.3	149648
S20		31	11917	Omicron	10.9 ± 1.6	14.5 ± 1.6	132311
S21		19.4	3855192	B.1	30 ± 4.1	66.3 ± 4.1	604979
S22		26	321567	B.1	13.3 ± 0.4	19.1 ± 0.4	174285
S23		21	2754192	B.1	26.4 ± 3.6	52 ± 3.6	474493
S24		23	3541238	B.1	24.7 ± 3.6	52.5 ± 3.6	479056
S25		27	226565	B.1	12.1 ± 0.3	14.2 ± 0.3	131398
S26		24	2541238	B.1	19.9 ± 3.5	41.2 ± 3.5	375945
S27		>45	NA	NA	0.95 ± 0.36	NA	NA
S28		>45	NA	NA	0.652 ± 1.68	NA	NA
S29		>45	NA	NA	0.953 ± 0.65	NA	NA

The clinical specimens were impedimetrically investigated using the SARS-CoV-2 immunobiosensor. Referring to the estimated calibration curve, the limits of detection and quantification were investigated (5.7 and 17 pg/mL). The samples were tested in relation to the positive control (PC, 50 ± 2 kΩ for its payload 85 pg/mL), and the negative control (NC, 1.563 ± 0.07 kΩ, the assay threshold) was included as well. The rT-RT‒PCR was used to validate the fabrication and performance of the immunobiosensor

RT‒PCR analysis is included for validation

### Selectivity test

A selective diagnostic method is necessary to discriminate the current SARS-CoV-2 from the other respiratory viruses that probably exist in the tested clinical samples. Therefore, different interferent viruses were used for selectivity testing on the fabricated immunobiosensors. In this test, different sensor chips were used for each chip for one of the nontarget virus strains. The sensor chips showed no significant response toward the interferent viruses, including influenza, hCoVs (OC43, NL63, and 229E), and MERS-CoV.

**Fig. 5:**
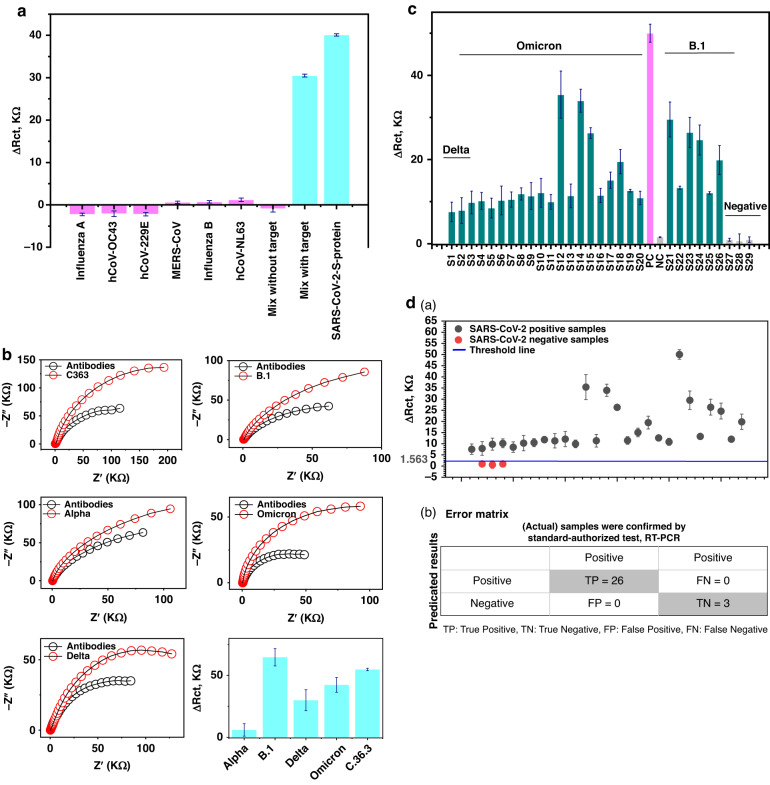
Selectivity testing of the biosensor performance towards non-targeting viruses as well as against different variants of the SARS-CoV-2. **a** Selectivity testing using different interferent respiratory viruses including Influenza, MERS-CoV, and other human coronaviruses. **b** Testing the immuno-biosensor for the discrimination of SARS-CoV-2 different variants including Alpha (B.1.1.7), B.1, Delta (B.1.617), C.36.3, and the circulating Omicron variant (BA.1). The sensor showed a significant response toward B.1 > C.36.3 > Omicron> Delta than Alpha variant. **c** Clinical sample application on 29 specimens. The positive (0.02 pg/mL) and the negative control were included in the experiment. The samples were representative of SAR-CoV-2 variants Delta, Omicron, and B.1 by 2, 18, and 6 samples, respectively. S# stands for the sample number. **d** (a) Classification of the investigated clinical specimens (positive and negative SARS-CoV-2 cases and confirmed by RT-PCR). (b) Error matrix of the positivity and negativity based on the threshold line (Threshold = 1.563) which is applied to 29 samples

As a confirmatory step, the sensor performance was tested against mixtures of interferent suspensions including and excluding the target antigen, where the sensor generated a notable electrochemical response only when the target virus strain was included in the suspension (Fig. 5a). Thus, the fabricated double-antibody-based immune biosensor was distinctly selective for SARS-CoV-2 in the tested specimens.

### Sensitivity of the immunobiosensor to SARS-CoV-2 variants

Recently, multiple variants of SARS-CoV-2 have been identified globally, with various mutations, especially in the RBD portion of the S protein^27,28^. The host immunogenic response to each RBD mutation-based S protein in the variants differed. Thus, the double-antibody-based immunobiosensor was tested for its response to isolated and identified variants, including Alpha, B.1, Delta, C.36, and the current globally circulating Omicron variants. The obtained impedimetric signals revealed that the degree of sensitivity of the fabricated sensors across the tested variant suspensions was 6.0, 65, 30, 42, and 55 kΩ (Fig. 5b). The efficiency of the newly designed biosensor to the identified variants could be assorted as follows: B.1 (100%)> Omicron (85%)>C.36 (66%)> Delta variant (47%) over Alpha variant (9.0%). The antigenic response of different variants, which have various antigenic drifts in the RBD portion toward the anti-RBD protective antibodies, was reported. For instance, higher reactive binding was observed to the Beta, Alpha, and Gamma variants than to the recent dominant variant (i.e., Omicron)^5^. Overall, the immunobiosensor chips based on a combination of antibodies to the RBD portion exhibits sensitive discrimination of SARS-CoV-2 variants.

### Reproducibility, repeatability, and accuracy

To cover all necessary sensing characteristics, sensor reproducibility, repeatability, and accuracy were tested. For this purpose, EIS responses were collected from several biosensor chips interacting with three different antigen concentrations (12.5, 13, and 15.5 pg/mL). As a result, high reproducibility was obtained with a relative standard deviation of ~3.0% (Supplementary data, Table S1). Moreover, the chips showed highly repeatable impedimetric signals and estimated values. Additionally, the accuracy of the biosensor performance was determined using five different spiked samples with various antigenic payloads of 12.5, 13.5, 15.5, 17.5, and 21.5 pg/mL for S1, S2, S3, S4, and S5, respectively. The obtained accuracy of the sensor chips’ performance for the spiked samples was approximately 98% (Supplementary data, Table S2).

### Analysis of clinical specimens

Whole virus particles of SARS-CoV-2 were isolated from most of the tested specimens, including nasopharyngeal, anal, sputum, blood, and urine specimens^29^. Nasopharyngeal swabs are the most reliable specimen of choice for virus isolation and identification. Therefore, twenty-nine nasopharyngeal specimens were collected from infected patients and applied to the fabricated immunobiosensor to validate the chips and test their performance. Twenty-six samples tested positive for SARS-CoV-2, and only three samples tested negative. Since the emergence of SARS-CoV-2 in late 2019, several known virus variants have circulated among countries. Thus, the twenty-six positive samples were representative of the most recent circulating variants (i.e., B.1, Delta, and Omicron). Two samples were classified as Delta, eighteen samples as Omicron-type, and six samples as B.1-type. The collected samples were directly applied to the fabricated biosensors, and within 15 min, impedimetric signals were obtained before and after the virus bound to the immobilized antibody fragments on the immune biosensor. Moreover, the samples were molecularly tested using RT‒PCR as a confirmatory validation protocol for the fabricated biosensors (Table 2).

The impedimetric signals of the chips were obtained before and after the incubation of the tested samples. From the obtained differences in the charge transfer resistances (Δ*R*_ct_) resulting from the selective binding affinity of the tested samples to the immunobiosensor chips, the virus payload was determined according to the designed standard calibration curve. Moreover, positive and negative controls were included in the experiment (Fig. 5C).

Most importantly, the fabricated ready-to-use chips showed different impedimetric signals related to the virus concentration/variant. The impedimetric signals from the eighteen samples of Omicron were as follows: 9.8 ± 2.7, 10.2 ± 2.0, 8.5 ± 2.4, 10.3 ± 3.4, 10.5 ± 1.8, 11.8 ± 1.4, 11.3 ± 3.2, 12.1 ± 3.4, 9.9 ± 1.7, 35.4 ± 5.6, 11.4 ± 2.8, 34 ± 2.7, 26.3 ± 1.3, 11.5 ± 1.7, 15.1 ± 1.9, 19.5 ± 2.9, 12.6 ± 0.3, and 10.9 ± 1.6 kΩ for S3, S4, S5, S6, S7, S8, S9, S10, S11, S12, S13, S14, S15, S16, S17, S18, S19, and S20, respectively. In correlation with the obtained signals, the antigenic payloads were determined to be 12.5 ± 2.7, 12.3 ± 2.0, 10.1 ± 2.4, 13 ± 3.4, 13.5 ± 1.8, 16 ± 1.4, 18.6 ± 3.2, 18.2 ± 3.4, 12.5 ± 1.7, 72.5 ± 5.6, 11.6 ± 2.8, 65 ± 2.7, 42.5 ± 1.3, 15 ± 1.7, 27 ± 1.9, 37.5 ± 2.9, 16.4 ± 0.3, and 14.5 ± 1.6 pg/mL, respectively. The fabricated biosensors showed a sensitive response to the Omicron variant-classified samples, and the PCR Ct values confirmed the results (Table 2). However, the chips were less consistent for the B.1-related samples, including S21, S22, S23, S24, S24, S25, and S26, where the obtained signals were 30 ± 4.1, 13.3 ± 0.4, 26.4 ± 3.6, 24.7 ± 3.6, 12.1 ± 0.3, and 19.9 ± 3.5 kΩ, respectively. Illustrating high performance of the fabricated chips, the antigenic concentrations of the virus were 66.3 ± 4.1, 19.1 ± 0.4, 52 ± 3.6, 52.5 ± 3.6, 14.2 ± 0.3, and 41.2 ± 3.5 pg/mL, respectively, and the impedimetric response was found to be correlated to the PCR results, with threshold lines at 19.4, 26, 21, 23, 27, and 24, respectively. However, the concentrations 7.5 ± 2.3 and 9.2 ± 3.1 pg/mL corresponded to 7.6 ± 2.3 and 7.9 ± 3.1 kΩ, respectively, for the 2 representative specimens for Delta variants (S1, S2), and these two samples were cut at the threshold line (CT) at 34 and 32, respectively.

Conversely, three samples were electrochemically classified as negative samples, where the resulting payload was below the estimated detection limit (i.e., 5.7 pg/mL) and were subsequently molecularly negative (Ct > 45).

Ultimately, the developed immunobiosensor was used to discriminate positive from the negative clinical samples based on thresholding values. The impedimetric response of the chips expressed in Δ*R*_ct_ was classified by the determination of a threshold value (=1.564), i.e., values above the threshold value were positive, while values and concentrations below the threshold were negative. Thus, as depicted in Fig. 5d-a, there were 26 positive samples and only three negative samples. Additionally, the classification of positive and negative samples is summarized in Fig. 5d-b, the error matrix in which the results are benchmarked to the standard authorized diagnostic test, RT‒PCR: the chips showed 100% specificity and sensitivity.

Therefore, according to the obtained response to the tested samples, the fabricated ready-to-use chips showed high performance and were sensitive to variants of concern (i.e., Omicron, B.1, and Delta). The quantitative analysis obtained by this biosensor from the extracted values of *R*_ct_ is of great value since the use of such disposable chips can enable both selective diagnosis and accurate determination of the antigenic payload of the virus in samples under investigation.

## Conclusion

The main advantages (the high specificity and sensitivity) provided by the newly fabricated SARS-CoV-2 immunobiosensor are supported by the use of a nanostructured sensor platform modified with AuNPs/WO_3_/CNTs, followed by the chemical immobilization of a combination of two antibodies. Accordingly, high electron transfer with minimal charge transfer resistances was obtained, as the AuNPs conjugate with the SAM layer of aminothiophenol and guarantee perfect arrangement on the nanocomposite base. The immobilization of both anti-S-RBD-SARS-CoV-2 and anti-Llama antibodies was carried out through covalent bonding with the exposed cross-linker chain. When assay optimization was achieved, high sensitivity and high selectivity were successfully obtained for both whole virus particles and purified S protein with limits of detection of 1.8 and 5.7 pg/mL, respectively. The chips showed highly selective performance for SARS-CoV-2 in the presence of other interfering viruses, including influenza and human coronaviruses and MERS-coronaviruses. Sensitive detection of most identified and circulating virus variants was observed, especially the Omicron and B.1 variants. For chip validation, 29 clinical specimens were tested, and the obtained impedimetric signals revealed that 18 samples were positive for the Omicron type, six samples were positive for B.1, two for Delta, and three samples were negative. The results were analyzed in comparison with RT‒PCR, and the results were reliable and representative of the virus’s presence by payload correlation according to the estimated calibration curve. The fabricated biosensor chips are suitable for the sensitive and selective detection of SARS-CoV-2 in the clinic without any sample preparation. The chips provide on-site detection and ready-to-use, quantifiable tools for virus screening.

### Supplementary information

Supplementary Data
