# Supplementary material for: Double-antibody-based nano-biosensing system for the onsite monitoring of SARS-CoV-2 variants
Source: Microsyst Nanoeng. 2023 Aug 21;9:105. doi: 10.1038/s41378-023-00578-0 (PMC10442362; doi:10.1038/s41378-023-00578-0)
Supplement: Supplementary file 1 — Supplementary Data [file 41378_2023_578_MOESM1_ESM.docx]

**Supplementary Data**

**Double-Antibody-based Nano-biosensing system for the Onsite Monitoring of SARS-CoV-2 Variants**

Heba A. Hussein^1,4^, Ahmed Kandeil^2^, Mokhtar Gomaa^2^, Rabeay Y. A. Hassan^3,4^*

^1^Virology Department, Animal Health Research Institute (AHRI), Agricultural Research Center (ARC), Giza, 12619, Egypt;

^2^Center of Scientific Excellence for Influenza Viruses, Environmental Research Division, National Research Centre, Giza 12622, Egypt;

^3^Nanoscience Program, University of Science and Technology (UST), Zewail City of Science and Technology, Giza 12578, Egypt;

^4^Biosensors Research Laboratory, Zewail City of Science and Technology, 6Th October City, Giza, 12578, Egypt

***Corresponding author**

Rabeay Y. A. Hassan

University of Science and Technology (UST),

Zewail City of Science and Technology,

6th October City, 12578 Giza, Egypt

Email: [ryounes@zewailcity.edu.eg](mailto:ryounes@zewailcity.edu.eg)

ORCID: <https://orcid.org/0000-0002-1867-9643>

**Table S1**: Reproducibility of SARS-CoV-2 immunobiosensor

| **SARS-CoV-2 Immunobiosensor Investigations (1^st^ & 2^nd^, reading)** | **S-protein Concentration (pg/mL)** | | | | | | | | |
| --- | --- | --- | --- | --- | --- | --- | --- | --- | --- |
|  | **12.5** | | | **13.5** | | | **15.5** | | |
|  | **1^st^** | **2^nd^** | **Mean±SD** | **1^st^** | **2^nd^** | **Mean±SD** | **1^st^** | **2^nd^** | **Mean±SD** |
| **ΔR_ct_ (R_ct_ _Antigen_-Rct _mAbs_), KΩ** | 12.7 | 13 | 12.8±0.2 | 13 | 13.6 | 13.3±0.4 | 13.6 | 13.7 | 13.6±0.08 |
| **Relative standard deviation, RSD%** | **1.8%** | | | **2.8%** | | | **0.6%** | | |

**Table S2**: Accuracy of SARS-CoV-2 immunobiosensor

| **Spiked samples** | **Actual virus payload**  **(pg/mL)** | **Estimated virus payload/ fabricated SARS-CoV-2 immunobiosensor (pg/mL)** | **Accuracy %** |
| --- | --- | --- | --- |
| **S1** | 12.5 | 12.4 | **100** |
| **S2** | 13.5 | 13 | **99** |
| **S3** | 15.5 | 15.8 | **98** |
| **S4** | 17.5 | 17.6 | **99** |
| **S5** | 21.5 | 21.6 | **100** |
